# 

**Published:** 1899-10-14

**Authors:** 


**MARRIAGE.**

STEPHENSON—DUCKITT.—On October 11th, at Horton Lane Congregational Chapel, Bradford, by the Rev. Henry Varley, B.A., assisted by the Rev. Samuel Griffiths, GEORGE BALME STEPHENSON to MARGARET DUCKITT, late Matron of the Eye and Ear Hospital, Bradford. At Home November 21st and 22nd. (466)

### NOTICE TO CORRESPONDENTS.

All MS., Letters, Books for Review, and other matters intended for the Editor should be addressed THE EDITOR, "THE HOSPITAL," THE HOSPITAL BUILDING, 28 & 29, SOUTHAMPTON STREET, STRAND, W.C.

The Editor cannot undertake to return rejected MS., even when accompanied by stamped directed envelope.

**Notice to Advertisers and Subscribers.**

All Advertisements, Orders for Copies of THE HOSPITAL, and Business Communications should be addressed to **THE MANAGER (Not to the Editor),** THE HOSPITAL, The Hospital Building, 28 & 29, Southampton Street, Strand, London, W.C.

The Cost of Subscription, post free, is as follows:—Per week, 2½d.; per quarter, 2s. 8d.; per half-year, 5s. 3d.; per annum, 10s. 6d. Foreign:—Per annum, 15s. 2d., payable, in all cases, in advance.

**NOTICE.—Vol. XXVI. of "The Hospital" (April, 1899, to September, 1899), in handsome cloth binding, will shortly be on sale at the Office of this Journal, price 7s. 6d. Cases for binding the Numbers contained in this Volume 1s. 6d. Index to Vol. XXVI., 2½d., post free.**

**The Monthly Part for October is now ready. Price 6d., by post 8d.**

### Scraps and Gleanings.

The amount collected this year by the Ilfracombe Hospital Saturday Fund amounted to £262, which has been handed to the Tyrrell Cottage Hospital.

The latest hospital scheme in Dollar proposes to purchase a property on the outskirts of the old town and convert it into an isolation cottage for fever patients. The purchase of the cottage in view would cost £415.

The Withington Council have applied to the Local Government Board for sanction to borrow £58,706 for the purpose of erecting a hospital for infectious diseases on the Baguley Lodge Estate, at Baguley, Cheshire. The Local Government Board have already given their sanction for the purchase of the site for such a hospital.

Since the foundation of the Royal Albert Orphan Asylum at Bagshot in 1864, 1,500 children have passed through the home, and over 200 at the present time are inmates of the asylum. Each boy or girl, it is found, costs the institution £21 7s. per annum, but if the interest on the mortgage be deducted that sum is reduced to £18 4s. 6d.

The amount collected this year by the Bolton Hospital Fund (upwards of £3,000) is £700 in excess of that obtained during the previous year, the expenses, however, being no heavier. The principal increase came from the engineering and iron foundry employés and card-room hands, who together had contributed nearly £400 more than last year.

The Medical Officer of Health for Settle, in a report to the Local Government Board on the accommodation provided at the Giggleswick Hospital for Infectious Diseases, condemns the existing premises, which he states should only be retained on condition that certain comprehensive alterations and additions be made, though even in such a case an up-to-date first-class hospital would not be obtained.

During the past year the amount contributed to the Dundee Royal Infirmary in subscriptions was £1,194 10s., whilst the working people contributed £2,406 2s. 8d. There is a deficiency in the year's income of £2,448, and, seeing that only some 340 persons last year contributed over one guinea to the infirmary, and only 140 persons gave subscriptions from 1s. to £1, it is clear that something more ought to be received in the way of small subscriptions.

The Perth Town Council have unanimously agreed to hand over to the Society of Incurables, for the purposes of the endowment of the proposed hospital for consumptives, the cost of which has been provided by Sir Robert and Lady Pullar, a sum of £392 12s. 11d., being the amount, with interest, raised in Perth in commemoration of the Diamond Jubilee towards the provision of an endowment fund for a hospital for consumptive patients. The remainder of the Diamond Jubilee Fund was (1) for local celebrations; and (2) for the Perth Sick Poor Nursing Society.

### LATEST TEXT-BOOKS FOR NURSES.

Crown 8vo., profusely Illustrated, cloth gilt, 5s.

### HANBOOK FOR NURSES.

By J. K. WATSON, M.D., M.B., C.M.

"A concise and comprehensive exposition of as much medical knowledge as a nurse needs. The work cannot but prove useful for whom it has been designed."—*Scotsman.*

New Edition, profusely Illustrated, Crown 8vo., cloth gilt, 3s. 6d.

### NURSING: Its Theory and Practice.

Being a complete Text-book of Medical, Surgical, and Monthly Nursing.
By PERCY G. LEWIS, M.D., M.R.C.S., L.S.A.
With entirely new Chapters on Mental Nursing, Mechanical Therapeutics, Massage, Schott, Tallerman, and Weir-Mitchell's Treatments.

"We have read the book with interest, and can warmly recommend it as a nursing manual."—*Birmingham Medical Review.*

Crown 8vo., Illustrated, cloth gilt, 7s. 6d. net.

### PRACTICAL POINTS IN NURSING.

For Nurses in Private Practice. By EMILY A. M. STONEY.

"The hints given are excellent, and we hope they may be widely read."—*British Medical Journal.*

Crown 8vo., profusely Illustrated, cloth gilt, 6s.

### PHYSIOLOGY: Experimental and Descriptive.

By B. P. COLTON, Professor of Natural Science, Illinois University.

Ready Shortly. Crown 8vo., Illustrated, cloth, 2s. 6d.

### NURSING IN DISEASES OF THE THROAT, NOSE, AND EAR.

By P. MACLEOD YEARSLEY, F.R.C.S.Eng., M.R.C.S., L.R.C.P.Lond.

*Complete Catalogue of Works on Nursing and allied subjects sent post free on application.*

London: THE SCIENTIFIC PRESS, Ltd., 28 & 29, Southampton Street, Strand, W.C.

### NOW READY,

A REPRINT OF

### A NURSING HANDBOOK

TOGETHER WITH S. MAW, SON, AND THOMPSON'S

### COMPLETE CATALOGUE OF NURSING REQUISITES.

The above work is now republished at a cost of 1s. per copy. Messrs. S. M., S., and T. have been compelled to make this charge on account of the extreme popularity of the work and the great expense incurred in its production.

***Post Free on receipt of 1s.***

### S. MAW, SON, and THOMPSON,

7 to 12, ALDERSGATE STREET, LONDON, E.C.

Nurses' Bags.

Clinical Thermometers.

### "THE BURDETT SERIES" OF POPULAR TEXT-BOOKS ON NURSING.

Small Crown 8vo., Cloth, Price 1s. each.

No. 1.—**PRACTICAL HINTS ON DISTRICT NURSING** By AMY HUGHES, late Superintendent of the Metropolitan and National Nursing Association.

No. 2.—**THE MATRON'S COURSE.** An introduction to Hospital and Private Nursing. By Miss S. E. ORME.

No. 3.—**THE MIDWIVES' POCKET-BOOK.** By HONNOR MORTEN. Author of "How to Become a Nurse, and How to Succeed," &c., &c.

No. 4.—**FEVERS AND INFECTIOUS DISEASES**: their Nursing and Practical Management. By WILLIAM HARDING, M.D.Ed., M.R.C.P.Lond., Author of "Mental Nursing," &c.

No. 5.—**NOTES ON PHARMACY AND DISPENSING FOR NURSES.** By C. J. S. THOMPSON.

No. 6.—**THE RATIONAL USE OF ANTISEPTICS IN MIDWIFERY PRACTICE.** By JAMES MORRISON, M.D., M.B., M.R.C.S., L.R.C.P.Lond. [*In preparation.*

No. 7.—**THE CARE OF CONSUMPTIVES.** By W. H. DAW, M.R.C.S., L.R.C.P.Lond.

No. 8.—**NURSING OF SICK CHILDREN.** By J. D. E. MORTIMER, M.B., F.R.C.S., L.S.A. [*Ready Shortly.*

No. 9.—**MENTAL NURSING.** By WILLIAM HARDING, M.D.Ed., M.R.C.P.Lond. [*Ready Shortly*

London: THE SCIENTIFIC PRESS, LTD., 28 & 29, Southampton Street, Strand, W.C.

